# The mechanical characteristics and performance evaluation of a newly developed silicone airway stent (GINA stent)

**DOI:** 10.1038/s41598-021-87142-w

**Published:** 2021-04-12

**Authors:** Hwa Sik Jung, Ganghee Chae, Jin Hyoung Kim, Chui Yong Park, Soyeoun Lim, Soon Eun Park, Ho Chang Kim, Young Jae Lee, Sung Kwon Kang, Don Han Kim, Yongjik Lee, Taehoon Lee

**Affiliations:** 1 Division of Respiratory and Critical Care Medicine, Department of Internal Medicine, Ulsan University Hospital, University of Ulsan College of Medicine, 877 Bangeojinsunhwan-doro, Dong-gu, Ulsan, 44033 Korea; 2 Department of Radiology, Ulsan University Hospital, University of Ulsan College of Medicine, Ulsan, Korea; 3 Department of Anesthesiology and Pain Medicine, Ulsan University Hospital, University of Ulsan College of Medicine, Ulsan, Korea; 4 Research and Development Department, S&G Biotech, Yongin-si, Gyeonggi-do Korea; 5 Department of Digital Contents Design, University of Ulsan College of Design and Architecture, Ulsan, Korea; 6 Department of Thoracic and Cardiovascular Surgery, Ulsan University Hospital, University of Ulsan College of Medicine, Ulsan, Korea

**Keywords:** Biological techniques, Biotechnology, Anatomy, Diseases

## Abstract

Central airway obstruction (CAO) can be attributed to several benign or malignant conditions. Although surgery is the preferred therapeutic option for the management of CAO, bronchoscopic treatment can be performed in scenarios where the surgical procedure is not possible. Recent years have witnessed several improvements in the field of bronchoscopic treatment, especially with regard to airway stents. Current research involves new attempts to overcome the existing shortcomings pertaining to the stents (migration, mucostasis, and granulation tissue formation). The authors have recently developed a new silicone airway stent (GINA stent) with an anti-migration design, dynamic structure that enables the reduction of stent cross-sectional area, and radio-opacity. The present study aimed to evaluate the mechanical characteristics and performance of the novel GINA stent using a porcine tracheal stenosis model. In the current study, all the tests involved the comparison of the GINA stent [outer diameter (OD, mm): 14; length (L, mm): 55] with the Dumon stent (OD: 14; L: 50). The mechanical tests were performed using a digital force gauge, in order to determine the anti-migration force, expansion force, and flexibility. The present study evaluated the short-term (3 weeks) performance of the two stents after implantation [GINA (n = 4) vs. Dumon (n = 3)] in the porcine tracheal stenosis model. The results pertaining to the comparison of the mechanical properties of the GINA and Dumon stents are stated as follows: anti-migration force (18.4 vs. 12.8 N, *P* = 0.008); expansion force (11.9 vs. 14.5 N, *P* = 0.008); and flexibility (3.1 vs. 4.5 N, *P* = 0.008). The results pertaining to the comparison of the short-term performance of the GINA and Dumon stents are stated as follows: mucus retention (0/4 vs. 0/3); granulation tissue formation (0/4 vs. 0/3); and migration (1/4 vs. 2/3). The GINA stent displayed better mechanical properties and comparable short-term performance, compared to the Dumon stent.

## Introduction

Central airway obstruction (CAO) is broadly defined as a blockage of the trachea, either main stem bronchus, and/or the bronchus intermedius, which might be attributed to several benign or malignant conditions^1^. Surgery (resection and anastomosis) could provide the best opportunity for definitive management. However, owing to the anatomical limitations, advanced and/or metastatic disease, or poor medical conditions, the surgical treatment of CAO is limited to certain benign conditions, such as tracheal stenosis after an infection, intubation, or tracheostomy. Eventually, in most cases, CAO can only be resolved by means of bronchoscopic interventions, which frequently require rigid bronchoscopy and include balloon dilatation, mass excision (debulking, debridement), tumor ablation (cryotherapy/argon plasma coagulation/electrocautery/laser), and stent insertion^2^. Although the resolution of CAO without using stents is desirable, in reality, the insertion of a stent is the primary technique employed in the treatment of CAO, owing to the cartilage damage in benign stricture or the externally compressive nature of the malignant stricture that occurs in most of the patients with CAO^3,4^.

Airway stents can be categorized into two main types, each with different characteristics. The metal stent has a strong expansion (radial) force, which has the advantage of a low migration rate. However, it is associated with a risk of granulation tissue overgrowth and tracheobronchial perforation. Conversely, the silicone stent has a low expansion force, thereby minimizing the risk of granulation tissue overgrowth and tracheobronchial perforation, whereas migration occurs easily. Mucus clogging affects both types of stents^1^. As such, airway stents are effective for immediate relief of CAO but causes complications such as migration, mucostasis, and granulation tissue formation frequently. Consequently, stent technologies are persistently evolving, in order to overcome the aforementioned shortcomings. The examples include drug-eluting stents (for the inhibition of granulation tissue formation), stents with new designs (for the inhibition of granulation tissue formation/migration), stents with hydrophilic coating on the inner surface (for the inhibition of mucostasis), radiopaque silicone stents, customized three-dimensional (3-D) printed stents, and bio-absorbable stents^2,3,5,6^.

The authors recently developed a new silicone airway stent (named “GINA stent”) based on the anti-migration design, with a flexible, dynamic structure to enable the reduction of stent cross-sectional area, in order to enhance the bronchial expiratory flow, as well as radio-opacity, so that the stent can be easily identified through radioimaging. The purpose of the present study was to introduce the GINA stent through the assessment of the mechanical properties and quality of performance in comparison with an established stent, the Dumon stent, via the evaluation of the anti-migration force, expansion force, and flexibility, along with the evaluation of the short-term (3 weeks) performance of the stents using a porcine tracheal stenosis model.

## Results

### Mechanical characteristics of the GINA stent

**Figure 1:**
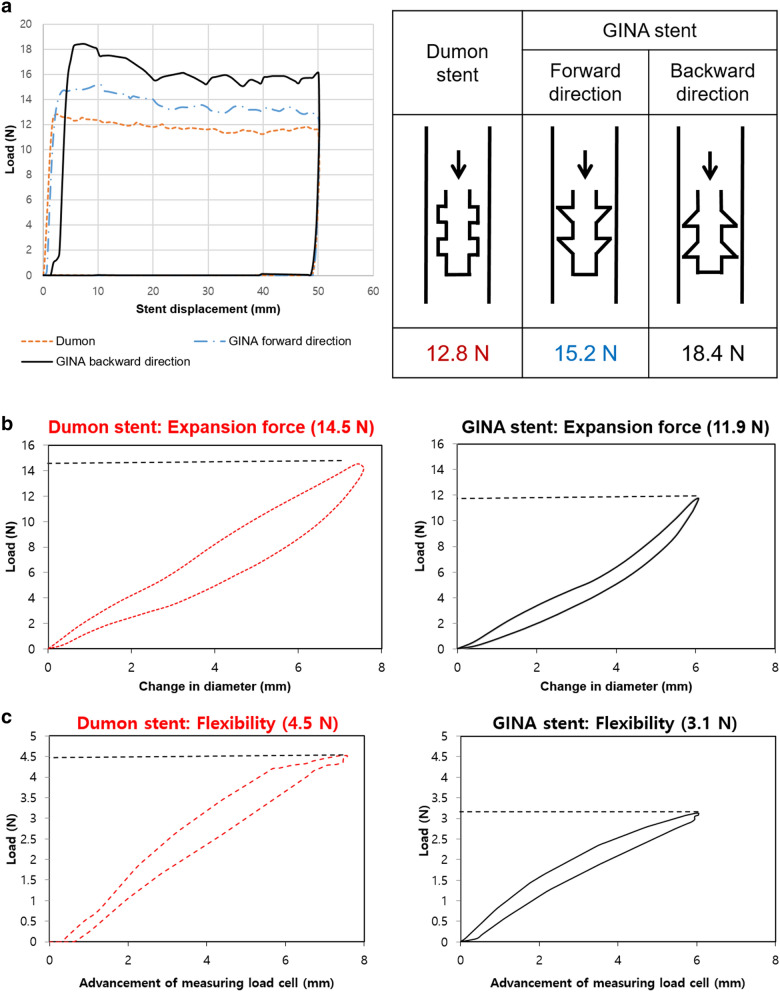
The results pertaining to the mechanical tests. (**a**) The GINA stent displayed stronger anti-migration force in both directions, compared to the Dumon stent. (**b**, **c**) The GINA stent displayed lower expansion force and higher flexibility. *N* Newton.

The anti-migration forces are illustrated in Fig. 1a and Supplementary Table 1. The GINA stent displayed stronger anti-migration forces in both directions, compared to the Dumon stent [Newton (N), mean ± Standard deviation (SD): Dumon stent, 12.83 ± 0.23 vs. GINA stent, 15.21 ± 0.59 (in the forward direction) and 18.4 ± 0.51 (in the backward direction), *P* = 0.008]. The stronger the anti-migration force, the lower the probability of stent migration, thereby minimizing the inherent complications associated with the stent.

**Table 1:** Short-term (3 weeks) performance of the GINA stent, compared to the Dumon stent, in a porcine model of tracheal stenosis.

Pig no.	Inner diameter of trachea on day 0, mm	Inner diameter of trachea on day 7, mm	Stenosis, %	Stent	Migration*	Time interval between stent insertion and migration, days	Mucus retention	Granulation tissue formation at both ends
1	17	11	58	GINA	No	–	No	No
2	18	12	56	GINA	No	–	No	No
3	16	10	61	GINA	Yes (proximal)	14	No	No
4	17	11	58	GINA	No	–	No	No
5	16	11	53	Dumon	Yes (proximal)	7	No	No
6	16	10	61	Dumon	Yes (proximal)	14	No	No
7	17	12	50	Dumon	No	–	No	No

*P = 0.486 for GINA versus Dumon.

The GINA stent displayed a lower expansion force, compared to the Dumon stent (N, mean ± SD: 11.91 ± 0.21 vs. 14.54 ± 0.27 N, *P* = 0.008) (Fig. 1b and Supplementary Table 1). Additionally, in terms of the stent flexibility, the GINA stent required a lower force to flex the stent, compared to the Dumon stent (N, mean ± SD: 3.13 ± 0.06 vs. 4.47 ± 0.10, *P* = 0.008) (Fig. 1c and Supplementary Table 1). Low expansion force and high flexibility can reduce the risk of granulation tissue formation and airway perforation. The results of the mechanical tests indicate that the GINA stent has superior mechanical properties, compared to the Dumon stent.

### Performance of the GINA stent in a porcine model

The results of the evaluation of short-term (3 weeks) performance of the GINA stent, compared to the Dumon stent, in a porcine model of tracheal stenosis are summarized in Table 1. Stent migration was detected on the fourteenth day after stent implantation in one of the four pigs that underwent GINA stent insertion. Among the three pigs that underwent Dumon stent insertion, stent migration was detected on the seventh and fourteenth day after stent insertion in two pigs. All the stent migrations were observed to be proximal migrations that were completely expelled from the body. Mucus retention and granulation tissue formation were not detected in either of the two types of stents during the 3 weeks after insertion (Fig. 4e, f).

## Discussion

GINA stent, a radiopaque silicone airway stent that was recently developed by the authors, was designed to minimize migration, mucostasis, and granulation tissue formation. The mechanical tests performed in the current study confirmed that the GINA stent has a lower possibility of migration and granulation tissue formation, compared to the Dumon stent. Moreover, the results of the performance evaluation using porcine models suggest that the performance of the GINA stent is not inferior to that of the Dumon stent.

The conditions regarding an ideal airway stent are cost-effectiveness, ease of insertion and removal, devoid of migration or granulation tissue formation, not excessive but adequate expansion force against airway stenosis, adequate flexibility to preserve the airway physiology, and without any impairment of the mucociliary clearance^7^. However, no stent is capable of fulfilling all of the aforementioned conditions; if one characteristic is superior, another tends to be inferior. For instance, if the stent has low expansion force and the likelihood of granulation tissue formation is reduced, the airway fixation of the stent declines, thereby increasing the risk of stent migration. Accordingly, metal stents have a low migration rate, but a high rate of granulation tissue formation, whereas silicone stents have a high migration rate, but a low rate of granulation tissue formation^1^. In terms of the inhibition of mucostasis in the stent, it is desirable to maintain the mucociliary clearance, and necessary to be flexible so that the stent inner diameter is sufficiently reduced during exhalation. The uncovered metal stent could best preserve the mucociliary clearance. However, removal of the stent is difficult, owing to epithelialization, in addition to the problem of tissue ingrowth within the stent. Consequently, an uncovered metal stent is not recommended for the management of benign airway stenosis; the use is restricted to the palliation of malignant airway stenosis, but tumor ingrowth should be a concern^8^. After all, improving the flexibility of the stent (enabling the reduction of stent cross-sectional area) is the most rational method of resolving mucostasis, thereby facilitating the removal of airway secretions through the enhanced expiratory flow.

In the present study, the GINA stent showed a lower expansion force and higher flexibility, compared to the Dumon stent. Although the current study did not observe any substantial difference between the two types of stents with regard to the formation of granulation tissue in the porcine models, it could be attributed to the short observation period. Granulation tissue formation is a common complication of silicone airway stents, although less than in metal stents^1,4,9^. Excessive expansion force and low flexibility are the predisposing factors associated with granulation tissue formation^9–12^. The low expansion force and high flexibility of the GINA stent implies that less force is required to expand and bend the stent, resulting in less pressure on the airway, leading to diminished mucosal inflammation and granulation tissue formation.

Despite the low expansion force, the GINA stent displayed a higher anti-migration force, compared to the Dumon stent, which was further confirmed by the animal experiments. The aforesaid superiority can be attributed to the creative surface design of the GINA stent, which comprises a right-angled triangle-shaped outer ring pertaining to the cartilaginous trachea and a raised, three-line arrangement pertaining to the membranous trachea. During the formulation of the design of the GINA stent, we reviewed several previously developed airway stents and the most inspiring were the Freitag stent and the Natural stent^13–15^. The two aforementioned stents have a common outer ring for cartilaginous trachea, which plays a role in the inhibition of migration. The current design improved on this feature (to maximize the anti-migratory friction) by transforming the outer ring into a right-angled triangle shape, and including a raised, three-line arrangement for the membranous trachea. The right-angled triangle-shaped outer ring is specifically designed to further suppress the proximal migration of the stent, which is more dangerous, compared to distal migration (proximal migration can lead to glottic obstruction or complete stent breakaway, resulting in suffocation)^16,17^. The present study confirmed this through the results of the mechanical test, which showed an improved anti-migration force in relation to the GINA stent backward direction. Moreover, the in-vivo effectiveness was recognized to a certain extent through the evaluation of short-term performance. Migration is a common complication associated with airway stents, especially silicone stent^18,19^, and several attempts have been made to inhibit the same. The Montgomery T tube was fabricated with a side arm that passes through a tracheostomy, which provides the stent with a fixation to the trachea^20^. Recently, an external fixation method was introduced, which resolved the cosmetic problem associated with the Montgomery T tube^21^. However, these methods can only be employed for the management of upper tracheal stenosis. In case of lower tracheal or bronchial stenosis, a bifurcation stent might facilitate the prevention of migration. However, the stent insertion is a challenging endeavor, owing to the size of the stent, which is excessively large, compared to the segment of stenosis^22,23^. Nevertheless, in order to prevent the migration of the stent, it is necessary to improve the friction (i.e., anti-migration force) using stents of suitable dimensions [diameter larger than the stenosis, but slightly smaller (80–90%), compared to the airway diameter around the stenosis]^4,24^ as well as by improving the stent surface design (like studs, spikes, or protruding arcs)^5,16,21,25,26^.

Another important feature of the GINA stent is the flexible, dynamic structure, which enables the reduction of the stent cross-sectional area. The GINA stent has a flat part, similar to the membranous portion of the actual tracheobronchial tree, which makes the stent more contractible and facilitates the removal of airway secretions through an enhanced expiratory flow. Freitag and Kim have already demonstrated that flattening a part of the stent improves the mucostasis^13–15,27^. The current performance study did not observe any substantial difference between the two types of stents with regard to the mucostasis, which might be ascribed to the short observational period.

Lastly, the GINA stent is radiopaque, which makes stent-tracking easier. The radiolucency of silicone stents (such as Dumon stents) has been considered to be a major drawback and efforts have been made to improve the same. Recently, a radiopaque version of the Dumon stent was developed.

Despite the success associated with the development of the GINA stent, the current study does not preclude limitations. First, the sample size of the animals that were involved in the experimental evaluation of the performance of the stents was small and the duration of observation was short. The current study did not observe any substantial difference between the two types of stents with regard to the mucus retention and granulation tissue formation, which is presumed to be due to the short duration of the experiment. The current study observed a difference between the two types of stents with reference to migration. However, it was not statistically significant, on account of the small sample size. Second, the current study did not compare the histology of the tissues at the sites of stent insertion. However, a follow-up study to ascertain the difference between the two types of stents with regard to the degree of injury at the site of stent insertion will be planned in the future. Third, the current study performed the mechanical tests on the basis of the advice provided by the stent manufacturing company (S&G Biotech, Gyeonggi-do, Korea) and previous studies^5,28,29^. Due to the lack of a validated method, existing studies have used simple or complex methods according to the nature and requirements of the respective studies. Consequently, the authors were compelled to conduct the experiments in a selective manner, in accordance with the laboratory conditions. For instance, it is more desirable to assess the anti-migration force using ex-vivo tracheal tissue or the materials that mimic the same, but we could not. Therefore, the current results should be interpreted with due consideration of the limitations.

In conclusion, the authors have developed a new stent through strategic design, which has reduced migration, despite the low expansion force and increased flexibility, in order to reduce the likelihood of granulation tissue formation. The scenario warrants future clinical trials to demonstrate the efficacy and safety of GINA stents in humans.

## Methods

### The GINA stent: a newly developed silicone airway stent

The GINA stent was fabricated using biocompatible silicone through the process of injection molding. It was intended to have an improved anti-migratory property and maintain airway patency while not exerting excessive force on airway wall. By referring the physical (mechanical) properties of the Dumon stent, the target force ranges of the expansion force and flexibility of the GINA stent were set to 10.0–13.5 N and 2.5–4.0 N, respectively, which are slightly lower (but not too low) than the Dumon stent's measurement (N, mean ± SD: 14.54 ± 0.27 and 4.47 ± 0.10, respectively). The target of the friction (anti-migration) force was aimed at more than 13 N, which is higher than that of the Dumon stent (N, mean ± SD, 12.83 ± 0.23) (Supplementary Table 1). Since the expansion force and flexibility themselves also affect the friction, we tried to ensure the friction force improvement was achieved through a strategic anti-migratory stent design. By way of repeated stent fabrication and physical measurements, the mixing ratio of silicone, barium, and hardener, and the heating temperature and time were variously modulated. Along with this, the stent surface design was also continuously adjusted. We finally developed a stent that meets our target force ranges.

**Figure 2:**
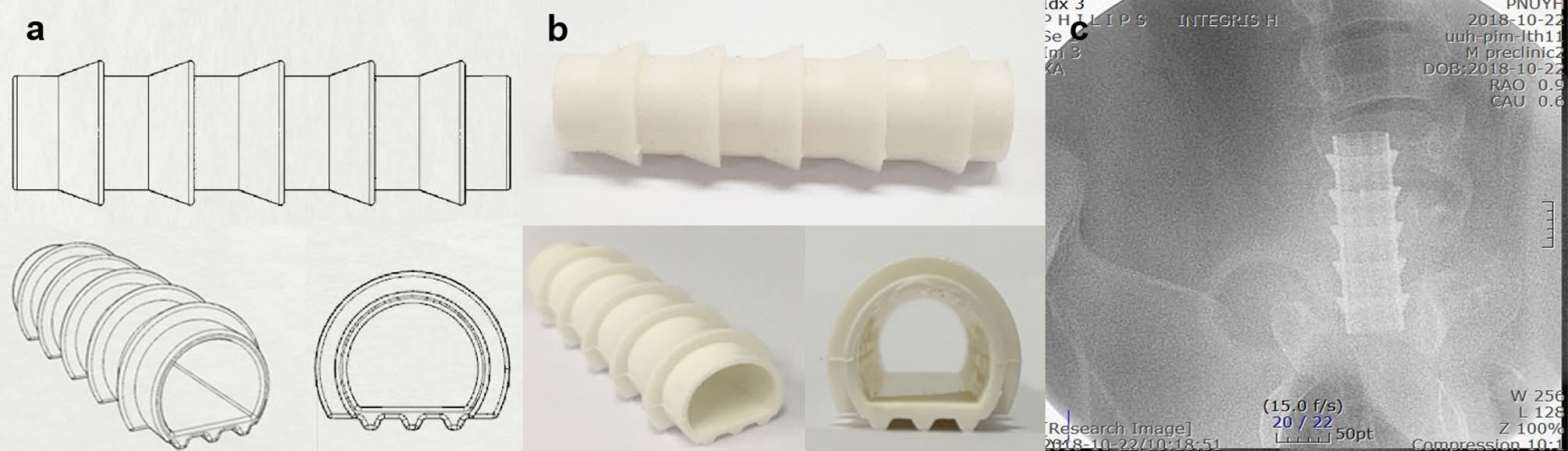
GINA stent, the silicone stent recently developed by the authors, has an anti-migration design, dynamic structure that enables the reduction of stent cross-sectional area, and radio-opacity. (**a**) Design of the GINA stent. (**b**) Actual GINA stent [outer diameter of 14 mm (OD14), ring diameter of 18 mm (RD18), and length of 55 mm (L55)]. (**c**) Radio-opacity of the GINA stent.

The GINA stent displayed three specific characteristics. First, the anti-migration design: the outer ring (right-angled triangle shape) of the GINA stent was designed to fit into the cartilaginous trachea; the plane of the stent that comes into contact with the membranous trachea was characterized by a raised, three-line arrangement. Second, a flexible, dynamic structure was considered, in order to facilitate the reduction of the stent cross-sectional area. GINA stent maintains the physiological airway contraction by means of the flat portion of the stent (similar to the membranous portion of the tracheobronchial tree), which renders greater flexibility and facilitates the removal of airway secretions through an enhanced expiratory flow. Third, radio-opacity: barium sulfate was added to the silicone to make the stent radiopaque, so that the stent can be easily identified by means of radiographic investigations (Fig. 2).

In the present study, all the assessments involved the comparison of the GINA stent with the Dumon stent (Novatech, France). A GINA stent with an outer diameter of 14 mm (OD14), ring diameter of 18 mm (RD18), length of 55 mm (L55), and a wall thickness of 1 mm and a Dumon stent with an outer diameter of 14 mm (OD14), surrounding studs of 2 mm (which makes the longest diameter of the Dumon stent 18 mm), length of 50 mm (L50), and a wall thickness of 1.5 mm were selected for the mechanical tests and animal study.

### Mechanical tests of the GINA and Dumon stents

Three mechanical properties (anti-migration force, expansion force, and flexibility), which indicate the actual physiological conditions, were evaluated. The anti-migration force is the frictional force generated between the stent and the airway. In general, the stronger the friction, the lesser the migration^30^. Hence, the frictional force was evaluated to estimate the anti-migration force. The expansion (radial) force is the radial force exerted by the stent on the airway. Flexibility is the elasticity of the stent in response to the external force (i.e., contraction of the airway). Excessive expansion force and low flexibility increase the friction, but could induce granulation tissue formation or airway perforation^9–12^. Therefore, in order to increase the friction, it is desirable to improve the stent design (which could inhibit migration) rather than increase the expansion force or decrease the flexibility. The present study directly evaluated the expansion force and flexibility, in order to assess the risk of granulation tissue formation and airway perforation. All the mechanical tests were performed by the Research and Development Department of the S&G Biotech (Gyeonggi-do, Korea)^31^ using the following methods. The forces were measured using a digital force gauge (LR5K Plus, Lloyd Instruments, Hampshire, England).

**Figure 3:**
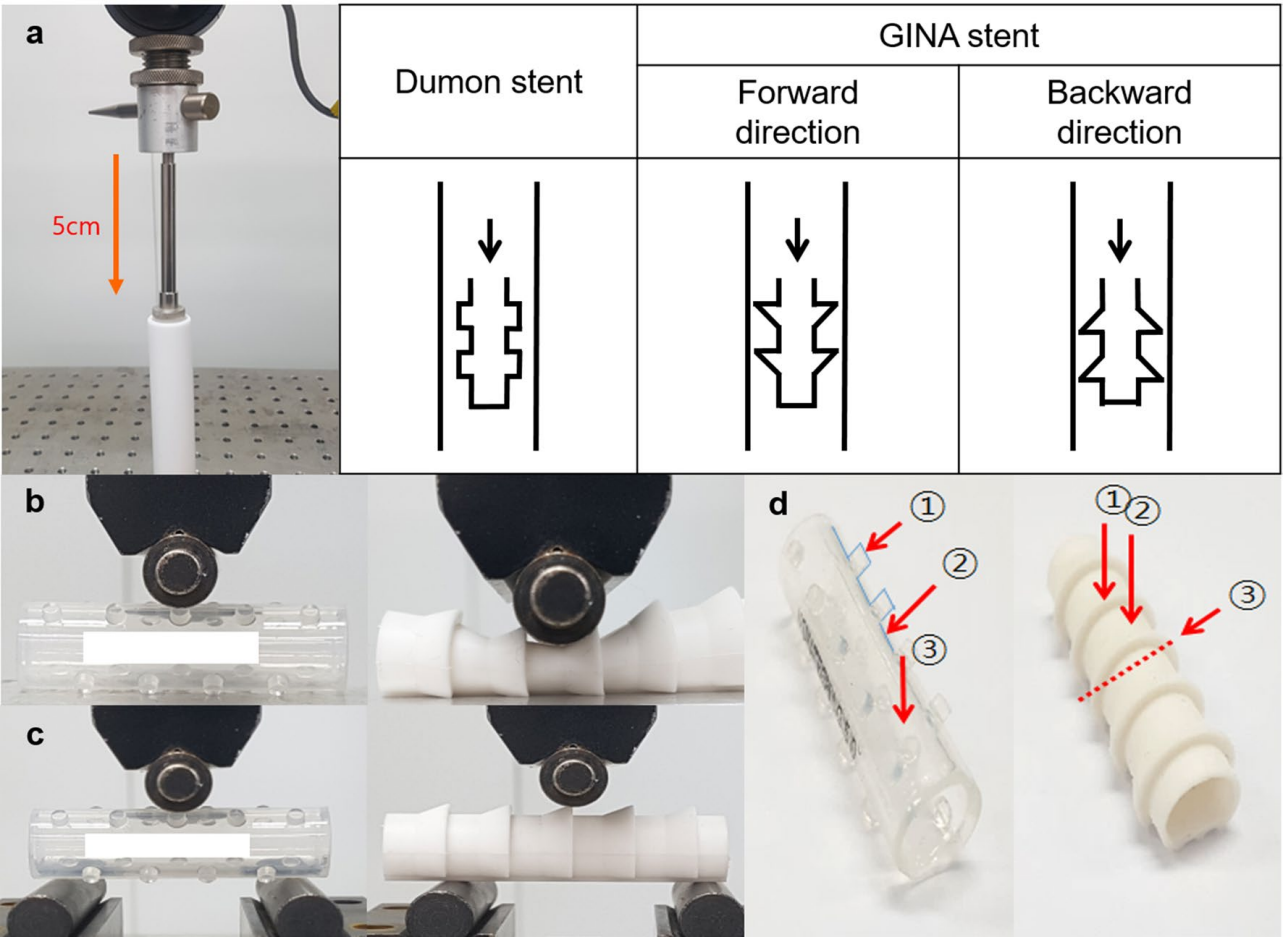
The methods used in the mechanical tests. The present study evaluated three mechanical properties (anti-migration force, expansion force, and flexibility) of the GINA and Dumon stents by means of a digital force gauge. (**a**) The anti-migration force was measured by pushing (5 cm) the stent through a Teflon jig. In the GINA stent, the test was performed in both the forward and backward directions. (**b**) The expansion force was evaluated by pushing the stent on a flat surface until the stent diameter was reduced by 50%. (**c**) Flexibility was assessed by placing the stent on separate jigs and pushing it by half the diameter of the stent. (**d**) The arrows indicate the different directions pertaining to the measurements of expansion force and flexibility.

The anti-migration force was evaluated using the following method: a Teflon jig with an inner diameter of 16 mm was fixed to the tensile strength tester. Subsequent to the insertion of the stent into the Teflon jig, a push gauge was used to move the stent to the opposite side of the Teflon jig by 5 cm. Concurrently, the anti-migration force was evaluated (Fig. 3a). The average of measurements (n = 5) was considered as the anti-migration force pertaining to each stent. In case of the GINA stent, the test was performed in both the forward and backward directions.

The expansion force was measured as follows: the stent was positioned on a flat floor. Subsequently, the measuring load cell was attached to the stent surface. The force was measured until the diameter of the stent was reduced by 50% (Fig. 3b). The force was measured in various directions (Fig. 3d) with tests (n = 5) in each direction and the average of the values was computed. The highest value among the measurements pertaining to different directions was considered as the expansion force of each stent.

The flexibility was evaluated as follows: the measuring jigs were separated at intervals of 4 cm. Subsequently, the stent was placed on the jigs and the measuring load cell was attached to the stent surface. The measuring load cell pushed the stent slowly and proceeded to half the diameter of the stent. The force was measured, in accordance with the advancement of the measuring load cell (Fig. 3c). The measurements were made in various directions (Fig. 3d) with tests (n = 5) in each direction and the average of the values was computed. The lowest value among the measurements pertaining to different directions was considered as the flexibility of each stent.

### Evaluation of the performance of GINA and Dumon stents in animal models

The present study assessed the short-term (3 weeks) performance of the GINA stent, compared to the Dumon stent, using a porcine model of tracheal stenosis. In order to perform this experiment, seven 12-week-old, female farm pigs (body weight: 40–45 kg) were selected, on account of the similarity between the porcine and adult human tracheal diameters (16–20 mm).

**Figure 4:**
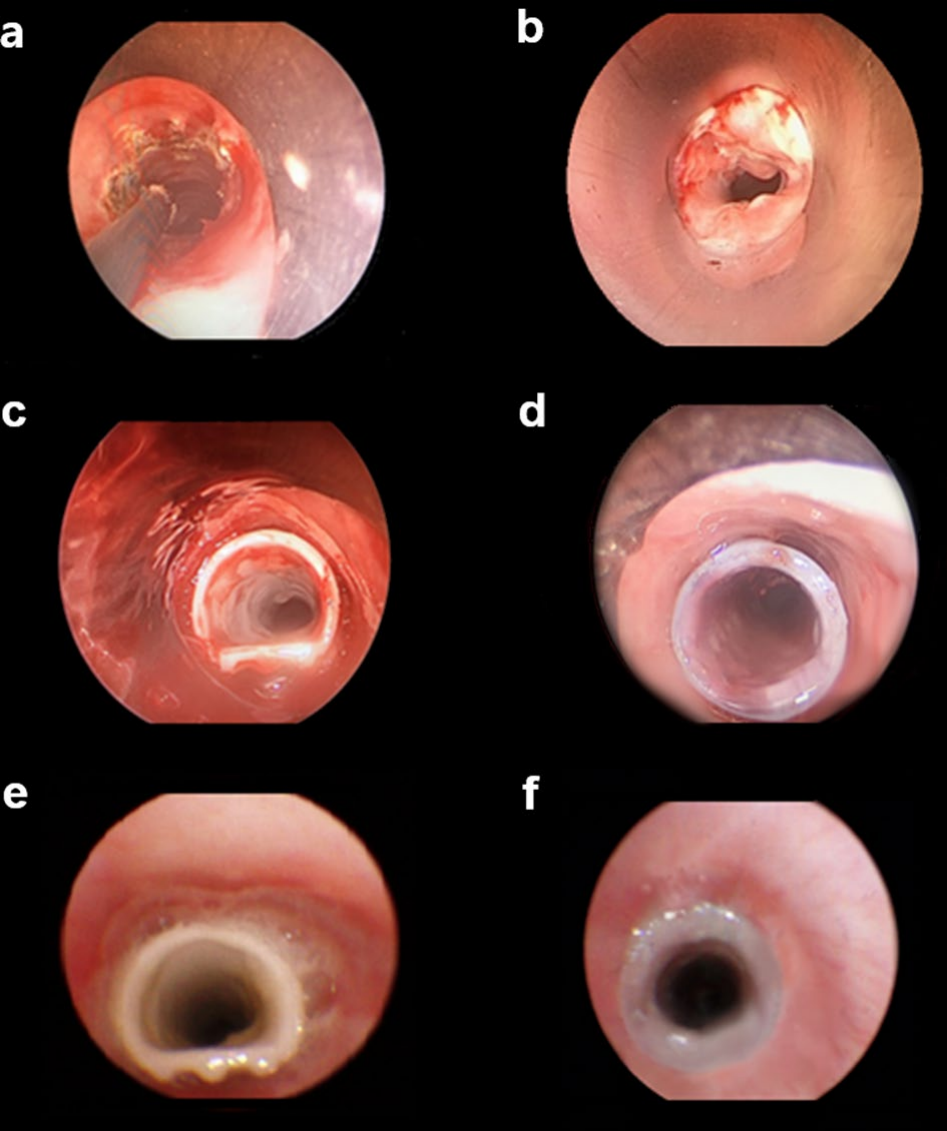
Evaluation of the performance of the GINA stent, compared to the Dumon stent, using porcine tracheal stenosis models. After inducing tracheal stenosis in 12-week-old pigs, a stent (GINA or Dumon) was inserted and the animals were observed for a time period 3 weeks. (**a**) The induction of stenosis (5 cm below the vocal cords) by means of cuff overpressure intubation and subsequent tracheal cautery. (**b**) Induced tracheal stenosis (a reduction in the measured airway cross-sectional area by at least 50%). (**c**, **d**) The GINA and Dumon stents inserted into the stenotic porcine tracheae. (**e**, **f**) The GINA and Dumon stents after 21 days.

Tracheal stenosis was induced 5 cm below the vocal cords using the methods of cuff overpressure intubation (COI) and tracheal cautery (TC), which were recently developed by the authors^32^. Momentarily, 200 mmHg of COI was applied to the porcine trachea by means of a silicone tracheal tube (internal diameter (ID): 9.0 mm; outer diameter (OD): 12 mm) for a duration of one hour. Subsequently, the tracheal mucosa in the region of the location of the cuff was cauterized [40 W by coagulation suction tube (10390 BN, Karl Storz, Germany)] by means of a rigid bronchoscope (size 8.5, 10318 BP, Karl Storz, Germany) (Fig. 4a). After 7 days of COI and TC, the induction of tracheal stenosis was confirmed by a ≥ 50% reduction in the measured airway cross-sectional area (Fig. 4b) and the stent insertion was performed.

Among the seven pigs, four received the GINA stent and three received the Dumon stent (Fig. 4c, d). A TONN/NOVATECH stent applicator (Novatech, France) was used to position the stents using a rigid bronchoscope (size 14, 10318 GL, Karl Storz, Germany). The stent performance was assessed by means of flexible bronchoscopy (MAF-TM, Olympus, Tokyo, Japan) on a weekly basis for a duration of 3 weeks after the stent insertion. The performance evaluation involved the examination of the stents to assess migration, granulation tissue overgrowth at both ends, and mucostasis.

In the present study, intramuscular alfaxan (5 mg/kg) and inhalational 3% isoflurane were used for the induction and maintenance of general anesthesia, respectively. All the experimental animals were handled in accordance with well-established bioethical guidelines (Guiding Principles in the Care and Use of Animals, DHEW Publication, NIH). The present study followed the protocol approved by the Institutional Animal Care and Use Committee of the Pusan National University Yangsan Hospital (Approval Number: PNUYH-2017-043), and was in compliance with the ARRIVE (Animal Research: Reporting of In Vivo Experiments) guidelines.

### Statistical analysis

The null hypothesis regarding the mechanical properties (anti-migration force, expansion force, and flexibility) was that there was no difference in the measurements between the two types of stents. The null hypothesis regarding the performance (migration, granulation tissue formation, and mucus retention) in a porcine model was that there was no difference in the incidences between the two types of stents. Statistical analyses were performed using SPSS 21 (IBM, Chicago, Illinois, USA). Because of the non-normal distribution and small numbers available for analyses, the Mann–Whitney U test (for continuous variables) and Fisher’s exact test (for dichotomous variables) were used to identify any potential associations. A *P* value < 0.05 was considered statistically significant in all analyses.

## Supplementary Information

Supplementary Table.

## Abbreviations

ARRIVE: Animal research: reporting of in vivo experiments
3-D: Three-dimensional
CAO: Central airway obstruction
COI: Cuff overpressure intubation
ID: Internal diameter
L: Length
N: Newton
OD: Outer diameter
RD: Ring diameter
SD: Standard deviation
TC: Tracheal cautery
