# Supplementary material for: The mechanical characteristics and performance evaluation of a newly developed silicone airway stent (GINA stent)
Source: Sci Rep. 2021 Apr 12;11:7958. doi: 10.1038/s41598-021-87142-w (PMC8041787; doi:10.1038/s41598-021-87142-w)
Supplement: Supplementary file 1 — Supplementary Table. [file 41598_2021_87142_MOESM1_ESM.docx]

**The mechanical characteristics and performance evaluation of a newly developed silicone airway stent (GINA stent)**

Hwa Sik Jung^1^, Ganghee Chae^1^, Jin Hyoung Kim^1^, Chui Yong Park^1^, Soyeoun Lim^2^, Soon Eun Park^3^, Ho Chang Kim^3^, Young Jae Lee^4^, Sung Kwon Kang^4^, Don Han Kim^5^, Yongjik Lee^6^, Taehoon Lee^1^

**Supplementary Table 1. Detailed results of the mechanical tests**

| Measurement No. | Anti-migration force (N) | | |  | Expansion force (N) | | | | | |  | Flexibility (N) | | | | | |
| --- | --- | --- | --- | --- | --- | --- | --- | --- | --- | --- | --- | --- | --- | --- | --- | --- | --- |
|  | Dumon | GINA forward | GINA backward |  | Dumon ① | Dumon ② | Dumon ③ | GINA ① | GINA ② | GINA ③ |  | Dumon ① | Dumon ② | Dumon ③ | GINA ① | GINA ② | GINA ③ |
|  |  |  | 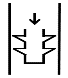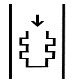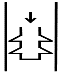 | 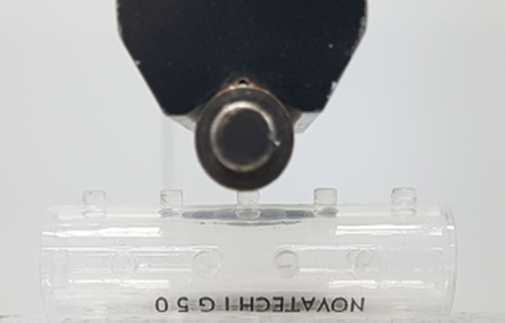   \|  \| \| --- \| |  | 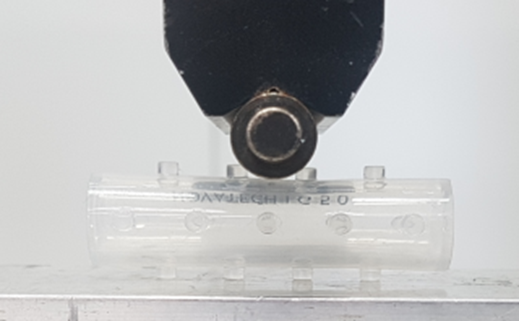 | 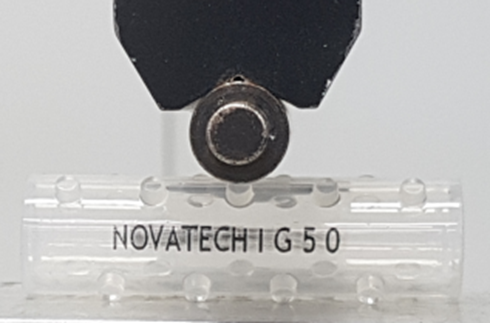 | 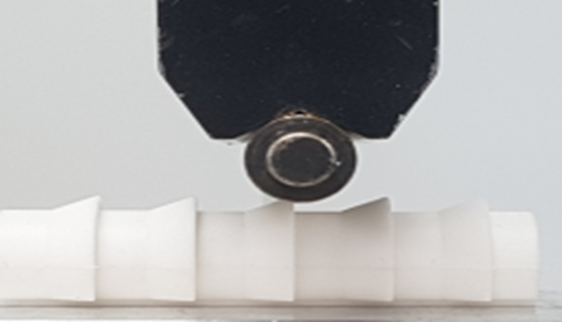 | 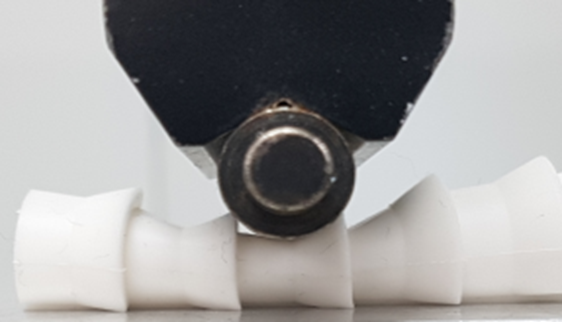 | 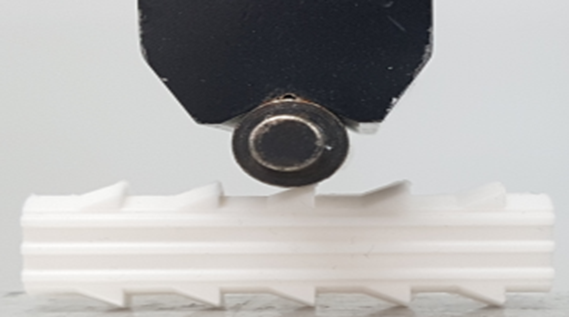 | 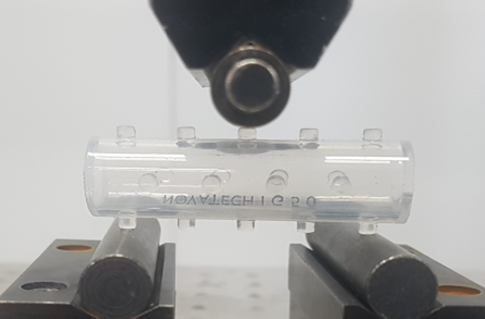   \|  \| \| --- \| |  | 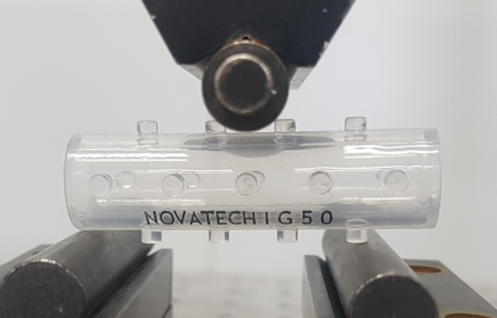 | 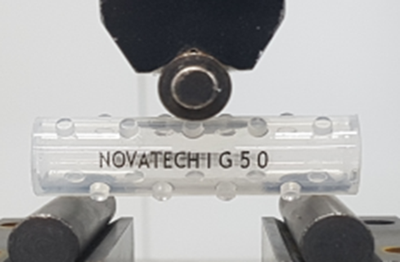 | 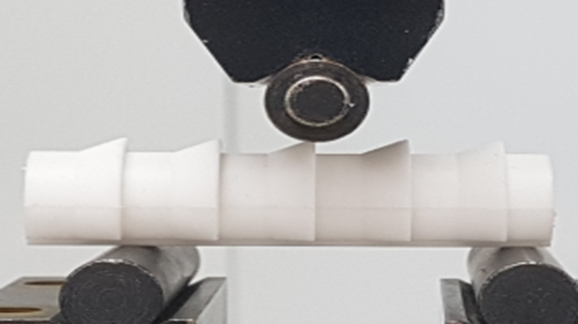 | 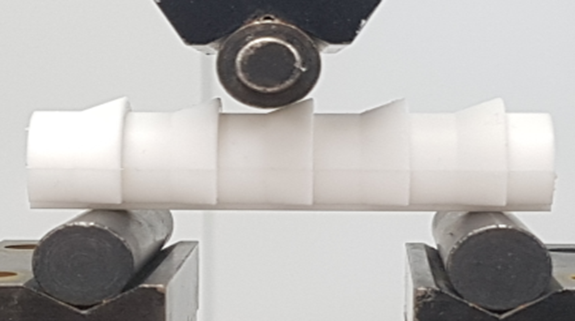 | 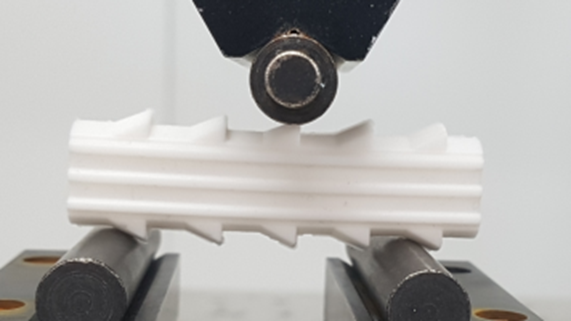 |
| 1 | 12.91 | 15.62 | 18.71 |  | 6.04 | 6.85 | 14.41 | 10.10 | 12.20 | 8.74 |  | 4.42 | 4.87 | 7.57 | 3.15 | 4.29 | 5.98 |
| 2 | 12.69 | 14.68 | 18.92 |  | 6.04 | 6.81 | 14.32 | 10.16 | 11.95 | 8.76 |  | 4.38 | 4.95 | 7.59 | 3.06 | 4.45 | 5.18 |
| 3 | 12.90 | 14.52 | 18.75 |  | 5.98 | 6.82 | 14.85 | 10.16 | 11.82 | 8.87 |  | 4.39 | 4.97 | 7.46 | 3.09 | 4.41 | 5.30 |
| 4 | 12.53 | 15.32 | 17.94 |  | 6.01 | 6.78 | 14.29 | 10.50 | 11.62 | 8.72 |  | 4.61 | 4.83 | 7.54 | 3.18 | 4.33 | 5.85 |
| 5 | 13.13 | 15.90 | 17.80 |  | 6.05 | 6.91 | 14.82 | 10.40 | 11.94 | 7.85 |  | 4.55 | 4.91 | 7.44 | 3.19 | 4.20 | 5.31 |
|  |  |  |  |  |  |  |  |  |  |  |  |  |  |  |  |  |  |
| Mean | 12.83 | 15.21 | 18.42 |  | 6.02 | 6.83 | 14.54 | 10.26 | 11.91 | 8.59 |  | 4.47 | 4.91 | 7.52 | 3.13 | 4.34 | 5.52 |
| Standard deviation | 0.23 | 0.59 | 0.51 |  | 0.03 | 0.05 | 0.27 | 0.18 | 0.21 | 0.42 |  | 0.10 | 0.06 | 0.07 | 0.06 | 0.10 | 0.36 |
| *P*-value |  | 0.008 (compared to Dumon) | 0.008 (compared to Dumon) |  |  |  |  |  | 0.008 (compared to Dumon ③) |  |  |  |  |  | 0.008 (compared to Dumon ①) |  |  |
| Abbreviation: N, Newton | | | | | | | | | | | | | | | | | |
